# Factors Associated With Gestational Diabetes Mellitus: A Cross-Sectional Study

**DOI:** 10.7759/cureus.17113

**Published:** 2021-08-11

**Authors:** Nazia Wagan, Adila Tahir Amanullah, Pushpa Bai Makhijani, Raveesha Kumari

**Affiliations:** 1 Obstetrics and Gynaecology, Sindh Government Qatar Hospital, Karachi, PAK; 2 Obstetrics and Gynaecology, Dow Medical College and Dr. Ruth K. M. Pfau, Civil Hospital Karachi, Karachi, PAK; 3 Obstetrics and Gynaecology, Tahir Medical Centre, Karachi, PAK; 4 Obstetrics and Gynaecology, Isra University, Hyderabad, PAK

**Keywords:** gestational diabetes mellitus, oral glucose tolerance test, fasting blood sugar

## Abstract

Introduction

The absence of tolerance in the levels of carbohydrates at the onset or at the time of pregnancy amongst females is known as gestational diabetes mellitus (GDM). This study is designed to determine the frequency of GDM and factors responsible for GDM to assess the actual magnitude of the outcome. Furthermore, it allows for developing strategies to minimize morbidities and improve the pregnancy outcome by early diagnosis and timely management, which can help reduce the frequency of GDM. The aim of the study was to determine the frequency of GDM and the factors responsible for GDM.

Methods

This was a cross-sectional study conducted in the Department of Obstetrics & Gynaecology Unit 2, Civil Hospital Karachi from the period starting from March 1, 2017, and ending on August 31, 2017, in order to determine the prevalence and associated risk factors of GDM. The study was carried out on 674 pregnant women. A total of 185 consecutive booked cases between the ages of 20 and 40 years, with parity 1 or more with gestational age greater than 24 weeks, were included in the study. Fasting plasma glucose levels 5.1-6.9 mmol/L (92-125mg/dl) and two hours plasma glucose levels of 8.5-11.0 mmol/L (153-199mg/dl) were set up as cut-off levels. GDM and factors were labeled on the basis of cut-off levels. Factors responsible for GDM included high maternal age when the age of the women was greater than 35 years and grand multiparity when women having a number of children greater than five, that is, women who have given birth five or more times. The collection forms were completed in the postpartum period. All information was obtained through the patient's clinical record and prenatal card. Initially, all variables were analyzed descriptively. To see the association of the groups, the chi-squared test (χ2 test) or Fisher's exact test was used. The level of significance used for the tests was 5%.

Results

The prevalence of gestational diabetes was 9.73% (95% CI: 8.53-12.64). The average age of the patients was 28.99 ± 4.34 years. The average pre-gestational BMI was 25.44 ± 2.74. Out of 185 women, 127 (68.65%) were less than or equal to 30 years of age. The average pre-gestational BMI was 25.44 ± 2.74, and average gestational age was 28.99 ± 2.34 years, respectively. A total of 161 (87.03%) of the women had a family monthly income of more than 10,000 PKR. There were 61 (32.97%) primiparous, 97 (52.43%) multiparous, and 27 (14.59%) grand multiparous women. Most of the women were illiterate numbering 36 (19.46%) or primary educated, numbering 30 (16.22%), and secondary educated or higher numbering 6 (3.24%). High maternal age (>30 years), high parity (>3), previous history of GDM, and family history of GDM were the significant factors of GDM.

Conclusion

The results of our study showed that the prevalence of gestational diabetes was 9.73% (95% CI: 8.53-12.64). Therefore, this study also showed that developing GDM was directly related to the following factors; such as the history of GDM in previous pregnancies with advanced maternal age, increased parity, and any medical history including a family history of GDM, along with the level of education of women. Hence, early detection and intervention are important because it improves pregnancy outcome.

## Introduction

The term "gestational diabetes" has also been used to describe glucose levels in early pregnancy that do not meet standard non-pregnant criteria for overt diabetes but are diagnostic for gestational diabetes [1, 2].

The identification of gestational diabetes mellitus (GDM) is vital for women in their pregnancy since it is often related to substantial changes in the metabolism of the pregnant women and also augments the chances of developing maternal morbidities and in some cases, even death [3].

The principal effects of GDM on pregnant women include delivery using the caesarian method, weight of birth beyond the 90th percentile, neonatal hypoglycemia, and levels of serum C peptide above the 90% level. The secondary effects of GDM include delivery that is considered premature or one that occurs before 37 weeks of gestation, chances of injury to the newborn (NB), the requirement to keep the NB in intensive care of the neonatal nature, hyperbilirubinemia, and preeclampsia [4].

Women suffering from GDM also experience resistance to insulin during their pregnancy, which is often accompanied by secretion of hormones including but not limited to growth hormones, hormones that are responsible for releasing corticotrophin, placental lactogen, and progesterone [5]. These changes combined with other alterations in the metabolism of a pregnant woman make sure that the fetus is supplied with nutrients that are ample in quantity. Pregnant women usually develop GDM when their pancreas is not able to counter the resistance of insulin which is linked with the state of pregnancy.

Although the range of developing GDM is from 1 to 25% for pregnant women in the United States, the rates of prevalence are generally around 5 to 6% [6] in India while the rate of GDM was 7.1% as reported by Rajput R et al. [7]. The rates of prevalence are not constant throughout the world and vary with regards to ethnicity and location being in line with the prevalence of type 2 diabetes. When we talk about the United States, the frequency of occurrence of GDM is generally higher among minorities when compared with the Caucasian population [8]. The rates of prevalence are also different for various other reasons which include different geographies of the population such as age and BMI, variation in the methods used for screening pregnant women, differences in the techniques used for testing, and variation in the criteria used for diagnosis [9].

The percentages of the occurrence of GDM have gradually increased with time, potentially due to an advanced average maternal age combined with increased weight or BMI of pregnant women [10, 11].

Women suffering from GDM during their pregnancy are also at a high risk of developing other problems including but not limited to fetal macrosomia, hypoglycemia, mental problems, chances of death for the NB, and various other problems. Therefore, it is essential to detect and treat GDM at an early stage to avoid other such morbidities and for ensuring the good health of the fetus of pregnant women. The goal of our study is to evaluate the prevalence of GDM combined with its linked factors of risk, in a sub-population of women in their pregnancy.

## Materials and methods

This was a cross-sectional study conducted in the Department of Obstetrics & Gynaecology Unit 2, Civil Hospital Karachi from the period of duration between March 1, 2017 and August 31, 2017. The sample size was calculated by taking the least proportion of 8.4% (5) sample size which was 185 pregnant women by using the OpenEpi website formula with a 95% CI and maximum error of ±4%. A total of 185 consecutive booked cases of ages between 20 and 40 years with parity 1 or more with gestational age (GA) greater than 24 weeks (weeks of pregnancy calculated on the basis of earliest available ultrasound scan) were included.

Those women who had not given consent and who had documented history of diabetes mellitus, hypertension, and endocrine disorder such as thyroid on treatment were excluded. Pregnant women who had NB diagnosed with malformation at the time of delivery were also excluded. Informed consent was taken before filling the proforma. The proforma was filled by a postgraduate trainee. Antenatal protocol showed that women with any factor attending the antenatal outpatient department (OPD) called at 24th week GA in OPD with the overnight fast of 6-8 hours; then fasting blood sugar was taken by reflectometer (capillary blood) using the oral glucose tolerance test (OGTT) with 75 grams of glucose dissolved in 200 ml of water then blood sugar taken after two hours by a reflectometer. Blood glucose level was diagnosed by using WHO criteria of OGTT [12]. Fasting plasma glucose levels 5.1-6.9 mmol/L (92-125mg/dl) and two hours plasma glucose levels of 8.5-11.0 mmol/L (153-199 mg/dl) were set up as cut-off levels. GDM and factors were labeled on the basis of cut-off level. Factors responsible for GDM included high maternal age when women's age was greater than 35 years and grand multiparity when women having a number of children is greater than five, that is, women who have given birth five or more times. An elevated maternal BMI as indicated by the WHO guidelines with regards to weight divided by height greater than 25, family history of 1st-degree diabetes mellitus such as in siblings or parents, and any prior history of GDM.

The collection forms were completed in the postpartum period. All information was obtained through the patient's clinical record and prenatal card. Initially, all variables were analyzed descriptively. For quantitative variables, this analysis was performed by calculating means and SDs. For qualitative variables, absolute and relative frequencies were calculated. To see the association between groups in relation to the proportions, the χ^2^ test or Fisher's exact test was used (when expected frequencies below five occurred). The level of significance used for the tests was 5%.

## Results

A total of 185 (n = 185) mothers were investigated, who gave birth between January 1 and December 31, 2017. Booked cases with greater than 24 weeks of GA of pregnancy were included in this study. The prevalence of gestational diabetes was 9.73% (95% CI: 8.53-12.64). Out of 185 women, 127 (68.65%) were equal to or less than 30 years of age, 28 (15.14%) were between 31 and 35 years of age, and 30 (16.22%) were above 35 years of age. The average age of the patients was 28.99 ± 4.34 years. The average pre-gestational BMI was 25.44 ± 2.74; the average height was 160.10 ± 3.69 cm; the average weight was 65.18 ± 6.77 kg and average GA was 28.99 ± 2.34 years, respectively. A total of 161 (87.03%) of the women had a family monthly income of more than 10,000 PKR. There were 61 (32.97%) primiparous, 97 (52.43%) multiparous, and 27 (14.59%) grand multiparous women. Most of the women were illiterate 36 (19.46%); primary educated were 30 (16.22%); secondary educated and higher educated were 6 (3.24%).

High maternal age (>30 years), high parity (>3), previous history of GDM, and family history of GDM were the significant factors of GDM while high BMI (>25 kg/m2) was not significant as shown in Table 1.

**Table 1:** Factors responsible for gestational diabetes mellitus. * = p-value less than 0.05 is significant. GDM: Gestational diabetes mellitus; Kg: Kilogram; m^2^: Meter square.

Factors	GDM		P-values	OR [95% CI]
	Yes [n = 18]	No [n = 167]		
High maternal age (>35 years)	9 (50%)	21 (12.6%)	<0.001*	4.30 [1.867-9.902]
High parity (>5)	6 (33.3%)	21 (12.6%)	0.018	2.44 [1.006-5.942]
High BMI (>25 kg/m^2^)	9 (50%)	71 (42.5%)	0.543	0.988 [0.414-2.357]
Previous history of GDM	12 (66.7%)	9 (5.4%)	<0.001*	20.1 [7.15-56.6]
Family history of GDM	18 (100%)	49 (29.3%)	<0.001*	0.836 [0.77-0.908]

Stratification analysis was performed and observed that the rate of GDM was not significant between two groups of the family monthly income of the women (p = 0.0624) while the rate of GDM was statistically significant with highly educated women. 

Significant factors for GDM include prior history of GDM including other factors like increased maternal age (more than 35 years), high parity (more than 5), lack of proper education, and low economic status with income less than 10,000 were significant factors for GDM as shown on Table 2.

**Table 2:** Comparison of gestational diabetes with demographics factors. * = p-value less than 0.05 is significant. GDM: Gestational diabetes mellitus.

Study characteristics	GDM		Total	P-value
	n = 18	n = 167	n = 185	
	Yes	No		
Family monthly income				
5,000 to 10,000	3 [17%]	21 [13%]	24 [13%]	0.624
>10,000	15 [83%]	146 [87%]	161 [87%]	
Education of women				
Illiterate	9 [50%]	104 [62%]	113 [61%]	<0.001*
Primary	6 [33%]	30 [18%]	36 [19%]	
Secondary	0 [0%]	30 [18%]	30 [16%]	
Higher	3 [17%]	3 [2%]	6 [3%]	

## Discussion

GDM represents a possible complication to which pregnant women are exposed. This disorder is defined as a state of glucose intolerance of varying degrees, detected the first time during pregnancy [13]. Typically, it is related to an increase in perinatal morbidity and mortality, as well as a greater frequency of long-term complications in the mother and her offspring, instances that would explain its correct identification and management [7].

A study conducted by Brody SC et al. [14] in 2003 showed that roughly 1 out of every 20 women in their pregnancy developed GDM which is in line with the conclusions from the study that we have carried out. Despite the results above, the chances of developing GDM in our sample size showed a percentage of around 9.73% (95% CI: 8.53-12.64) which is in excess of the prevalence documented among other populations in Turkey which generally ranged between 1 and 5% [15]. These discrepancies may probably have to do with the differences in the sizes of the samples and the mean age of the population under consideration. Further analysis of the available literature highlights the fact that chances of developing GDM may further be increased with increased mean age and a family history of GDM in the past.

The association observed between GDM and family history of diabetes mellitus is in agreement with the findings of the literature, being part of the risk factors listed by various consensuses [16-18]. Moreover, physicians seem to have different opinions with regards to the time duration during which screening for GDM may occur. For example, according to the recommendations of the WHO, it is suggested that all women in their pregnancy should be tested for GDM between weeks 24 and 28 [19]. On the other hand, the American Diabetes Association suggests that women with advanced age or those who are at high risk of developing GDM should be screened in the first trimester while the rest of the pregnant women may be tested in the same way as recommended by the WHO [1, 20].

As well as family history of diabetes mellitus and age, a higher BMI is also considered a classic risk factor, being cited in all consensuses. This study found that adjustments for family history of diabetes mellitus, and education, as well as for variables of the same level, 0.836 (0.77-0.908) decreased the estimated risk of having GDM among overweight women. Similarly, high maternal age (>35 years), high parity (>5), and previous history of GDM may increase the risk of GDM (OR = 4.30 [1.867-9.902]), (OR = 2.44 [1.006-5.942]), and (OR = 20.1 [7.15-56.6]), although it still remains highly significant.

In the study done by Dahanayaka NJ et al. [21] in 2012, a total of 405 pregnant women were recruited. Out of which the prevalence of GDM was found to be in 8.9% and the same factors were responsible for GDM such as 78 (19.3% ) were high BMI (>25 kg/m2) and 34 (8.4%) were of high maternal age (>35 years), which is very similar to our study. Worldwide, in pregnant women, GDM affects an estimated 15% [22]. Similarly, another meta-analysis study has been published by Lee KW et al. in 2018. The findings of this study showed that the significant factors of risk for GDM included prior history of GDM (OR: 8.42; 95% CI: 5.35-13.23), BMI in excess of 25 (OR 3.27; 95% CI 2.81-3.80), and hypertension caused by pregnancy (OR: 3.20; 95% CI: 2.19-4.68) among other similar factors indicated by our study [16].

For women suffering from GDM, the rate of maturity for the fetal lungs generally takes longer and the average age of gestation for the pulmonary maturation falls between 34 and 35 weeks. Almost all fetuses take maturity by the 37th week. However, for pregnant women with GDM, the lungs may not reach maturity until about 39 weeks. Secondly, it is vital for pregnant women undergoing induction to have a cervix that is ripe; if not so there is an increased risk of failure with regards to the induction that may eventually end up in resulting a caesarian section.

In summary, this study showed that the estimated risk of GDM is associated with having a high maternal age and having a family history of diabetes mellitus. Direct effect on the estimated risk of GDM was observed in association with maternal age, education, high parity (>5), high BMI (>25 kg/m2), and previous history of GDM was directly associated with GDM. Family history of GDM in the first six months of pregnancy had a protective effect against GDM.

Recommendation

Pregnant women above the age of 35 with a BMI in excess of 25 are recommended to be screened for GDM. Since pregnant women with GDM are at a high risk of developing complications before, during, and after pregnancy, it is essential to provide excellent levels of care to these women so that the chances of developing birth-related complications may be decreased and the overall results may improve. There is a need to carry out more studies to indicate those populations that seem to be at a higher risk for developing GDM. Physical exercise and exertion before and during pregnancy may decrease the chances of developing glucose tolerance that is not considered normal. Thus, in addition to being over 25 years of age, excessive central fat deposition, obesity or excessive weight gain in ongoing pregnancy and a family history of diabetes mellitus should be included among the risk factors for GDM.

## Conclusions

The results of our study showed that the prevalence of gestational diabetes was 9.73% (95% CI: 8.53-12.64). Therefore, this study also showed that developing GDM was directly related to the following factors such as a history of GDM in previous pregnancies with advanced maternal age, increased parity, and including medical history like family history of GDM including education of women. For pregnant women, GDM is related to elevated levels of morbidities in both the mothers and the fetus. It is vital to screen and detect GDM in the initial stages and intervene accordingly in order to improve outcomes that are associated with the pregnancy. The identification of new risk factors for the development of GDM is important for planning future prevention strategies.

## References

[1] American Diabetes Association (2004). Gestational diabetes mellitus. Diabetes Care.

[2] Buchanan TA, Xiang AH (2005). Gestational diabetes mellitus. J Clin Invest.

[3] Samad N, Hassan JA, Shera AS, Maqsood A (1996). Gestational diabetes mellitus--screening in a developing country. J Pak Med Assoc.

[4] Metzger BE, Lowe LP, Dyer AR (2008). Hyperglycemia and adverse pregnancy outcomes. N Engl J Med.

[5] Zhu Y, Zhang C (2016). Prevalence of gestational diabetes and risk of progression to type 2 diabetes: a global perspective. Curr Diab Rep.

[6] Moyer VA (2014). Screening for gestational diabetes mellitus: U.S. Preventive Services Task Force recommendation statement. Ann Intern Med.

[7] Rajput R, Yadav Y, Nanda S, Rajput M (2013). Prevalence of gestational diabetes mellitus & associated risk factors at a tertiary care hospital in Haryana. Indian J Med Res.

[8] Ferrara A (2007). Increasing prevalence of gestational diabetes mellitus: a public health perspective. Diabetes Care.

[9] Behboudi-Gandevani S, Amiri M, Bidhendi Yarandi R, Ramezani Tehrani F (2019). The impact of diagnostic criteria for gestational diabetes on its prevalence: a systematic review and meta-analysis. Diabetol Metab Syndr.

[10] Dabelea D, Snell-Bergeon JK, Hartsfield CL, Bischoff KJ, Hamman RF, McDuffie RS (2005). Increasing prevalence of gestational diabetes mellitus (GDM) over time and by birth cohort: Kaiser Permanente of Colorado GDM Screening Program. Diabetes Care.

[11] Abouzeid M, Versace VL, Janus ED, Davey MA, Philpot B, Oats J, Dunbar JA (2014). A population-based observational study of diabetes during pregnancy in Victoria, Australia, 1999-2008. BMJ Open.

[12] López Stewart G (2014). Diagnostic criteria and classification of hyperglycaemia first detected in pregnancy: a World Health Organization Guideline. Diabetes Res Clin Pract.

[13] Jiménez-Moleón JJ, Bueno-Cavanillas A, Luna-Del-Castillo JD, Garciá-Martín M, Lardelli-Claret P, Gálvez-Vargas R (2002). Prevalence of gestational diabetes mellitus: variations related to screening strategy used. Eur J Endocrinol.

[14] Brody SC, Harris R, Lohr K (2003). Screening for gestational diabetes: a summary of the evidence for the U.S. Preventive Services Task Force. Obstet Gynecol.

[15] Erem C, Cihanyurdu N, Deger O, Karahan C, Can G, Telatar M (2003). Screening for gestational diabetes mellitus in northeastern Turkey (Trabzon city). Eur J Epidemiol.

[16] Lee KW, Ching SM, Ramachandran V (2018). Prevalence and risk factors of gestational diabetes mellitus in Asia: a systematic review and meta-analysis. BMC Pregnancy Child.

[17] Duman NB (2015). Frequency of gestational diabetes mellitus and the associated risk factors. Pak J Med Sci.

[18] Li Z, Cheng Y, Wang D, Chen H, Chen H, Ming WK, Wang Z (2020). Incidence rate of type 2 diabetes mellitus after gestational diabetes mellitus: a systematic review and meta-analysis of 170,139 women. J Diabetes Res.

[19] (2013). WHO: Diagnostic criteria and classification of hyperglycaemia first detected in pregnancy. World Health Organization.

[20] Li Y, Ren X, He L, Li J, Zhang S, Chen W (2020). Maternal age and the risk of gestational diabetes mellitus: a systematic review and meta-analysis of over 120 million participants. Diabetes Res Clin Pract.

[21] Dahanayaka NJ, Agampodi SB, Ranasinghe OR (2012). Inadequacy of the risk factor based approach to detect gestational diabetes mellitus. Ceylon Med J.

[22] Ginige S, Wijeyaratne K, Wijeyaratne CN (2011). Prevalence of gestational diabetes mellitus in Homagama Divisional Director of Health Service area. J Coll Community Physicians Sri Lanka.

